# Social comparison modulates the neural responses to regret and subsequent risk-taking behavior

**DOI:** 10.1093/scan/nsy066

**Published:** 2018-08-24

**Authors:** Zhiyuan Liu, Li Zheng, Lin Li, Jialin Xu, Xuemei Cheng, Xiuyan Guo, James Mulcahy, Min Xu

**Affiliations:** 1 School of Psychology, Shaanxi Normal University, Xi'an, China; 2 Shanghai Key Laboratory of Magnetic Resonance, School of Physics and Materials Science, East China Normal University, Shanghai, China; 3 School of Psychology and Cognitive Science, East China Normal University, Shanghai, China; 4 Key Laboratory of Brain Functional Genomics, Ministry of Education, Shanghai Key Laboratory of Brain Functional Genomics, School of Psychology and Cognitive Science, East China Normal University, Shanghai, China; 5 National Demonstration Center for Experimental Psychology Education, East China Normal University, Shanghai, China; 6 College of Mechanical and Electrical Engineering, Beijing Polytechnic, Beijing, China; 7 Department of Neuroscience, Brighton and Sussex Medical School, Brighton, UK

**Keywords:** regret, risk taking, social comparison, functional connectivity, ventral striatum, dACC

## Abstract

The current functional magnetic resonance imaging fMRI study investigated how outcomes achieved by others affect subjective regret and subsequent behavior. During the task, participants were asked to open a series of boxes consecutively until they decided to stop. Each box contained a reward (gold), except for one that contained an adverse stimulus (devil), which caused the participants to lose all the gold they collected in that trial. Importantly, participants were instructed that every trial they encountered would also be played in parallel by another player. During the feedback stage, outcomes of both the participant and the other player were presented. Behaviorally, participants felt less regret and took less risk when objective outcomes improved or when their outcomes were better than others. Participants tended to take more risk after experiencing regret. At the neural level, the ventral striatum (VS) and the pregenual anterior cingulate cortex (pgACC) showed increased activation as objective outcomes improved. Across participants, activation of the VS was positively correlated with corresponding behavioral changes. Increased activation of the VS and significantly higher functional connectivity with the dorsal anterior cingulate cortex (dACC) were found when their outcomes were better than others. Additionally, the VS–dACC functional connectivity was correlated with risk-taking behavior.

## Introduction

Individuals are faced with countless decisions every day. Often by selecting one option, one must also reject the alternatives. When the outcomes of these alternative options become known, this information can modulate the evaluation of the obtained outcome, a phenomenon known as counterfactual thinking (Roese, 1994; Roese and Olson, 1997; Zeelenberg *et al.*, 1998). Counterfactual thinking requires one to compare ‘what is’ with ‘what might have been’ (Bell, 1982; Zeelenberg, 1999; Zeelenberg and Pieters, 1999). Regret can be the product of counterfactual thinking, as it can be induced by the revelation that a better alternative outcome could have been obtained had another choice been made (Bell, 1982; Loomes and Sugden, 1982; Markman *et al.*, 1993; Connolly and Zeelenberg, 2002). Individuals tend to avoid such negative feelings by changing future decision behaviors, a process termed as regret-based learning (Loomes and Sugden, 1982; Brassen *et al.*, 2012). Theoretical (Foster and Vohra, 1999; Coricelli and Rustichini, 2009, 2010) and empirical (Coricelli *et al.*, 2005; Marchiori and Warglien, 2008) studies have shown that regret has an adaptive function—it constitutes a way of evaluating past outcomes to optimize future decisions. For example, in a sequential risk-taking task, Liu *et al.* (2016) found that participants who experienced regret due to risk aversion in the current trial tended to take more risks in the subsequent trial.

Previous studies on regret have predominantly induced regret by comparing actual and alternative outcomes of one individual without accounting for the decision-making of others. However, in everyday life, individuals are constantly surrounded by information about other people, for example, their performances and possessions, which can lead to the comparison of one’s own outcome and outcomes achieved by others. A number of studies have investigated the effects of social comparison on decision-making and emotions (He, 1997; Kumar, 2004; Hoelzl and Loewenstein, 2005; Bault *et al.*, 2008; Linde and Sonnemans, 2012; Habib *et al.*, 2015). For instance, in Kumar (2004), participants who were told that their friends had chosen their forgone alternative and received a greater discount tended to report less intention to stick to their purchase choice. Moreover, empirical results also indicate that social comparison influences the risk aversion of people who had experienced gain in a prior choice (He, 1997). Therefore, it may be reasonable to infer that the outcomes achieved by others could affect the experience of regret and regret-based learning.

The task originally used by Mellers *et al.* (1997) has been frequently adopted to study regret and relief (Camille *et al.*, 2004; Bault *et al.*, 2008; Habib *et al.*, 2015). In this task, participants are asked to make a choice between two alternatives. After the decision, outcomes of both selected and unselected alternatives are presented. In the studies that have employed this task, participants experienced regret when they won less or lost more than the unselected alternative. Conversely, they felt relief when their decision provided the greatest reward. Simple stand-alone decision-based tasks do not, however, accurately represent the complex decision-making processes individuals frequently face. People are often required to make many sequential risky decisions, for example, deciding when to sell stock. To address this issue, Brassen *et al.* (2012) employed a modified version of the sequential risk-taking task (Balloon Analog Risk Task) that could also induce regret effectively (Lejuez *et al.*, 2002; Rao *et al.*, 2008). During the task, participants were asked to open a series of eight boxes consecutively until they decided to stop. Each box contained a reward (gold), except for one that contained an adverse stimulus (devil), which caused the participants to lose all the gold they collected thus far in that trial. By using this sequential risk-taking task, researchers found that the striatum, the reward-related brain region (Knutson *et al.*, 2001, 2003; Schultz, 2002; O'Doherty *et al.*, 2003; Koeneke *et al.*, 2008; Izuma *et al.*, 2008, 2010; Haber and Knutson, 2010) was involved in the experience of regret. More specifically, activation of the striatum decreased as regret level increased (Brassen *et al.*, 2012; Liu *et al.*, 2016; Liu *et al.*, 2017). Moreover, a significant amount of evidence from reinforcement learning has revealed that the ventral striatum (VS) plays a central role in reward-based learning, demonstrated by adjusting behaviors in order to maximize rewarding or minimize aversive outcomes (Delgado, 2007; O'Doherty *et al.*, 2007; Schultz, 2007; Niv and Montague, 2008; Daniel and Pollmann, 2014). It has recently been suggested that functional connectivity between the VS and the dorsal anterior cingulate cortex (dACC) may play an important role in risk-taking behaviors (Steinberg *et al.*, 2008; Crone and Dahl, 2012; Porter *et al.*, 2015). Porter *et al.* (2015) proposed that motivation processed by the VS influences the dACC activity, with implications for the propensity of adolescents to display risk-taking behavior.

In the current study, by using the sequential risk-taking task, we predict that participants might feel less regret and will take less risk when obtained outcomes improve or when they perform better than others. At the neural level, based on previous findings (Fliessbach *et al.*, 2007; Schultz, 2007; Niv and Montague, 2008; Brassen *et al.*, 2012; Daniel and Pollmann, 2014; Liu *et al.*, 2016; Liu *et al.*, 2017), firstly, we hypothesise that the VS will show increased activation when obtained outcomes improve and that activation of the VS will be associated with regret-related behavior adjustments. Moreover, as Bault *et al.* (2011) demonstrate that the VS encoded social rewards, we secondly predict that when participants perform better than others, increased activation of the VS will be observed. In addition, we predict that the functional connectivity between the VS and other brain regions, such as the dACC, may change as a function of social comparison. The functional connectivity between the VS and the dACC might be related to subsequent risk-taking behavior.

## Experimental procedure

### Participants

Thirty right-handed participants (15 female, aged 19 to 26, *M* = 22.93, s.d. = 2.13) from the university community with normal or corrected-to-normal vision participated in this experiment. None of the participants had abnormal neurological history, and all gave informed consent before scanning. This study was approved by the Ethics Committee of East China Normal University.

### Procedure

Before scanning, participants were told that they would undertake a sequential decision-making task while undergoing functional magnetic resonance imaging (fMRI) scanning. Participants were instructed that every trial they encountered in the game would also be completed, in parallel, by another player and that both of the outcomes of their own and the other player’s game would be presented during the outcome stage. Participants were informed that the other player was the same gender as them and was also from the university community. Participants were also informed that payment for their participation would be affected by their gains from the task.

**Fig. 1:**
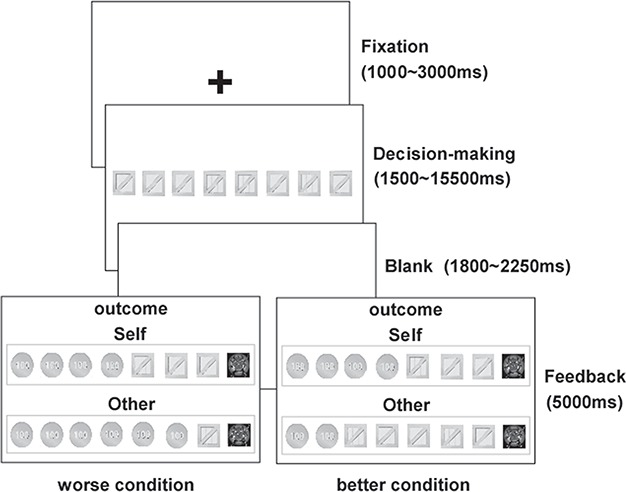
Two possible conditions are displayed when participants play the task during fMRI scanning. Participants decide to stop after collecting four gold coins. At the outcome stage, another player’s outcome was also presented. In both outcomes, the devil was presented in the same position. The outcome of another player might be better than, equal to or worse than the participants’. For example, the graph on the bottom left means worse condition, and the graph on the bottom right means better condition.

All participants completed 90 trials in the scanner. On each trial, an array of eight boxes was presented, where seven boxes contained gold coins and one box contained a devil. The position of the devil was randomised for each trial. Boxes were always opened from left to right. At any stage, participants had 2000 ms to either open the next box or stop and collect the gains acquired so far in that trial by pressing a key. Opening the box with the devil ended the current trial, and all gains from that trial were lost. A jittered interval (ranging from 1800 to 2250 ms) was presented after the participant decided to stop or after unpacking the devil. If participants stopped and collected gains, the actual position of the devil was revealed, thus informing participants about both the amount of gold they gained and the amount of gold they missed. During the outcome stage, another player’s outcome was presented along with the participant’s own outcome. In both outcomes, the devil was presented in the same position. Outcomes were highlighted on the screen by a cyan square (in the case of stopping and collecting the gains, i.e. Gain trial) or a red square (in the case of unpacking the devil and losing the gains in that trial, i.e. Loss trial). The outcome of the other player might be better than, equal to or worse than the participants’. The outcome was presented for 5000 ms. Finally, an additional jittered inter-trial interval (ranging from 1500 to 15 500 ms) was introduced. Figure 1 displays two of the possible outcome conditions for a trial.

After scanning, participants were presented with their own and the other player’s results from the task completed inside the scanner and were asked to rate how they felt, for each trial, on a 9-point scale from extreme regret (defined as −4) to extreme relief (defined as 4).

## fMRI data acquisition

Scanning was carried out on a 3 T Siemens Trio system at the Functional MRI Laboratory, East China Normal University, Shanghai. For functional images, 35 slices were acquired using a gradient-echo echo-planar imaging (EPI) sequence (Repetition Time (TR)  = 2200 ms, Echo Time (TE)  = 30 ms, Field of View (FOV) 10 = 220 mm, matrix size = 64 × 64, slice thickness = 3 mm, gap = 0.3 mm). Prior to fMRI measurements, a high-resolution structural image was acquired using a T1-weighted, multiplanar reconstruction (MPR) sequence (TR = 1900 ms, TE = 3.42 ms, 192 slices, slice thickness = 1 mm, FOV = 256 mm, matrix size = 256 × 256).

Data pre-processing and statistical analyses were performed using Statistical Parametric Mapping (SPM12, Wellcome Department of Cognitive Neurology, London).The functional images were corrected for the delay in slice acquisition and were realigned to the first image to correct for interscan head movements. The individual T1-weighted, 3D structural image was co-registered to the mean EPI image generated after realignment. The co-registered structural image was then segmented into gray matter, white matter and cerebrospinal fluid using a unified segmentation algorithm (Ashburner and Friston, 2005). The functional images, after slice timing and realignment procedures, were spatially normalized to the Montreal Neurological Institute (MNI) space (resampled to 2 ^*^ 2 ^*^ 2 mm^3^) using the normalization parameters estimated during unified segmentation and then spatially smoothed with a Gaussian kernel of 8 mm full-width half-maximum.

## Data analyses

### Behavioral data analyses

Before data analyses, we calculated a combined index called real gain percentage (RGP), which was defined as the ratio of the collected gain and the largest possible gain (that is, the total number of boxes before the devil) in a given trial (Liu *et al.*, 2016; Liu *et al.*, 2017). The value of the RGP can be considered an indication of how good the outcome was on a particular trial. The Gain conditions were then divided into three levels according to the value of the RGP: (i) Low RGP (LRGP, poor outcome, 15.1 ± 5.4 trials): 0 < RGP < = 0.6; (ii) Middle RGP (MRGP, moderate outcome, 19.4 ± 4.3 trials): 0.6 < RGP < = 0.8; and (iii) High RGP (HRGP, optimal outcome, 17.5 ± 4.8 trials): 0.8 < RGP < = 1. The division points between conditions were set *post hoc* to make trial numbers in each condition as similar to each other as possible.

According to the comparison between participants and others’ outcomes, three kinds of conditions could be defined in Gain trials: (i) worse condition (15.0 ± 4.0 trials), in which the collected gains of participants were less than that of the others; (ii) same condition (17.8 ± 4.1 trials), in which the collected gains of participants were the same as that of the others; and (iii) better condition (19.2 ± 3.0 trials), in which the collected gains of participants were larger than that of the others. In addition, Loss trials could be divided into two conditions: (i) worse condition (27.8 ± 1.8 trials), in which participants unpacked the devil and lost coins but others did not; and (ii) same condition (10.1 ± 3.2 trials), in which both participants and others unpacked the devil and lost coins.

Previous research has shown that emotional ratings in the current trial could predict behavioral changes in the next trial. Such results were only found in the Gain_Gain condition [trials in which participants did not unpack the devil (i.e. gain, collected golds) in both the current and the next trials] (Büchel *et al.*, 2011; Liu *et al.*, 2016). Therefore, in the current study, to investigate the behavioral changes between the current trial and the next, we restricted the analyses to the Gain_Gain condition. The behavioral changes between the current trial and the next were defined as the difference in the number of boxes being opened between two successive trials (Dif):Dif = Opened Boxes_*t*+1_ − Opened Boxes_t_ (Liu *et al.*, 2016).

### fMRI data analyses

To test the hypotheses, the current study conducted four models to analyze fMRI data.

Model 1 aimed to assess how brain activity was modulated by obtained outcome by performing a parametric analysis. The RGP level (LRGP, MRGP and HRGP) in the Gain trials and the number of lost coins in the Loss trials were used as parametric regressors. For this analysis, the conditions were time locked to the presentation of the outcome of the final decision, convolved with a canonical hemodynamic response function (HRF). Additional regressors included in the design matrix comprised the duration of decision-making phase and six movement-related parameters (three translation and three rotation parameters). High-pass temporal filtering with a cutoff of 128 s was also applied in the models. The resulting subject-specific estimates of the parametric regressors at each voxel were then entered into a second-level one-sample *t*-test.

In model 2, a parametric analysis was preformed to assess how brain activity was modulated by social comparison (worse, equal and better). The outcomes of social comparison (worse, equal and better) were used as parametric regressors. The remaining analysis was the same as that used in model 1.

In model 3, a parametric analysis was preformed to investigate the relationship between brain regions and emotional rating. Here, the emotional ratings in Gain and Loss trials were used as parametric regressors. The remaining analysis was also the same as that used in model 1.

Model 4 investigated how functional connectivity across brain regions associated with regret processing varied along different levels of social comparison using a psycho-physiological interaction (PPI) analysis (Friston *et al.*, 1997; O’Reilly *et al.*, 2012). We firstly used the peak voxels of right VS (MNI 8 18 2), identified in the second-level analysis (i.e. right VS showed increased activation from worse condition to better condition), to serve as a landmark for the individual seed voxels. For each participant, we searched within a 6 mm sphere around the coordinates of right VS from the second-level analysis to determine their individual peak voxels (a voxel-level threshold of P < 0.05). One participant did not show any activation within the sphere at the current threshold and was excluded from the analysis. For the remaining 29 participants, the time series that was extracted from a 6 mm-sphere drawn around the individual activation peaks, served as the physiological variable. The PPI analysis was then carried out (psychological variable 1 for better condition, −1 for worse condition) for each participant, and a design matrix was created with the interaction term, the psychological variable and the physiological variable as regressors. Participant-specific contrast images of the interaction term were entered into a second-level random-effects analysis using a one-sample *t*-test.

For all analyses, a cluster-level threshold of *P* < 0.05 Family-wise error (FWE) and a voxel-level threshold of *P* < 0.0001 (uncorrected) were used to define activations.

## Results

### Behavioral results

**Fig. 2:**
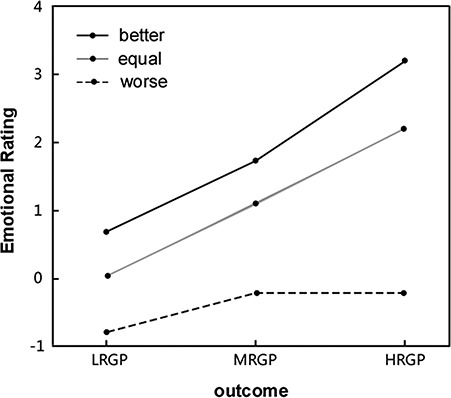
Emotional ratings were plotted as a function of social comparison (worse, equal and better) and outcome (LRGP, MRGP and HRGP). Further regression analyses showed participants felt less regret either when result of social comparison were positive (from worse to better condition) or when objective outcomes improved (from LRGP to HRGP outcome).

Firstly, we plotted emotional ratings for each condition, describing how emotional ratings might change as a function of social comparison (worse, equal and better) and outcome (LRGP, MRGP and HRGP) (Figure 2). Then, in order to investigate how social comparison and outcome affected the emotional ratings in Gain trials, a regression analysis was performed. In the regression analysis, social comparison, outcome and the interaction between them were included as predictors, and emotional ratings were used as the dependent variable. We used R-square to select the model that fitted the data best. The result showed that the model containing social comparison and outcome fit the data best (Table 1). The regression analysis revealed that emotional ratings could be explained by social comparison (β = 0.83, *P* < 0.001) and outcome (β = 1.16, *P* < 0.001) significantly. Both descriptive and regression analyses showed that participants felt less regret (more relief) either when the result of social comparison was more positive (from worse condition to better condition) or when objective outcomes improved (from LRGP outcome to HRGP outcome).

**Table 1:** Model selection

Predictors	R-square
Outcome	41.74%
Social comparison	37.64%
Outcome and social comparison	54.76%
Outcome, social comparison and	
outcome × social comparison	53.81%

Secondly, to investigate how social comparison and outcome in the current trial predicted behavioral changes (Dif) in the Gain_Gain condition, a regression analysis was performed, defining both social comparison and outcomes as the independent variables and behavioral changes (Dif) as the dependent variable. The result showed that the model containing social comparison and outcome fit the data best. The regression analysis revealed that emotional ratings could be explained by both social comparison (β = −0.56, *P* < 0.001) and outcome (β = −0.31, *P* < 0.001) significantly. This revealed that participants tended to take more risks in the subsequent trial when they were inferior to the other player or when they received only small objective gains in the current trial.

**Fig. 3:**
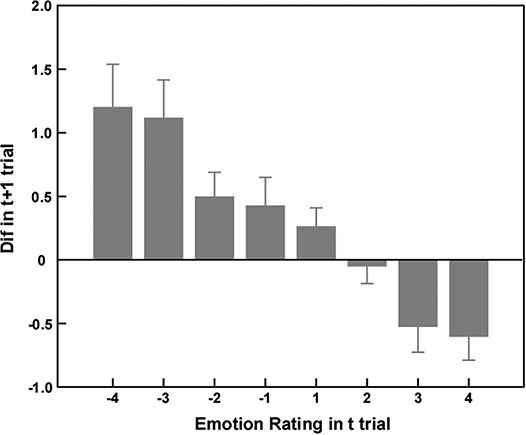
The relationship between emotional ratings in the current trial and behavioral change between the current trial and the next (Dif) in Gain_Gain condition. Further regression analyses revealed that the more regret participants experienced in the current trial, the more risks they would take in the next trial.

We next aimed to replicate our previous finding that emotional ratings in the current trial influenced behavioral changes in the next trial (Dif) in the Gain_Gain condition (Liu *et al.*, 2016). Therefore, another regression analysis was performed. Emotional ratings were defined as the independent variable, and the inter-trial behavioral change (Dif) was defined as the dependent variable. The results showed that emotional ratings in the current trial could significantly predict the behavioral change in the next trial (β = −0.20, *P* < 0.001). This result indicated that if participants experienced regret due to risk aversion in *t* trial, they tended to take more risk in *t* + 1 trial. Figure 3 describes how emotional ratings in the *t* trial influenced behavioral changes in the *t* + 1 trial.

Finally, another regression analysis was performed to investigate how social comparison and lost coins affected the emotional ratings in loss trials. Both social comparison and lost coins were defined as independent variables. The regression analysis revealed that emotional ratings could be significantly explained by both social comparison (β = 0.78, *P* < 0.001) and lost coins (β = −0.37, *P* < 0.001). This indicated that participants felt more regret when they lost more coins or when they did worse than the other player.

### fMRI results

#### The effect of social comparison in Gain trials

**Fig. 4:**
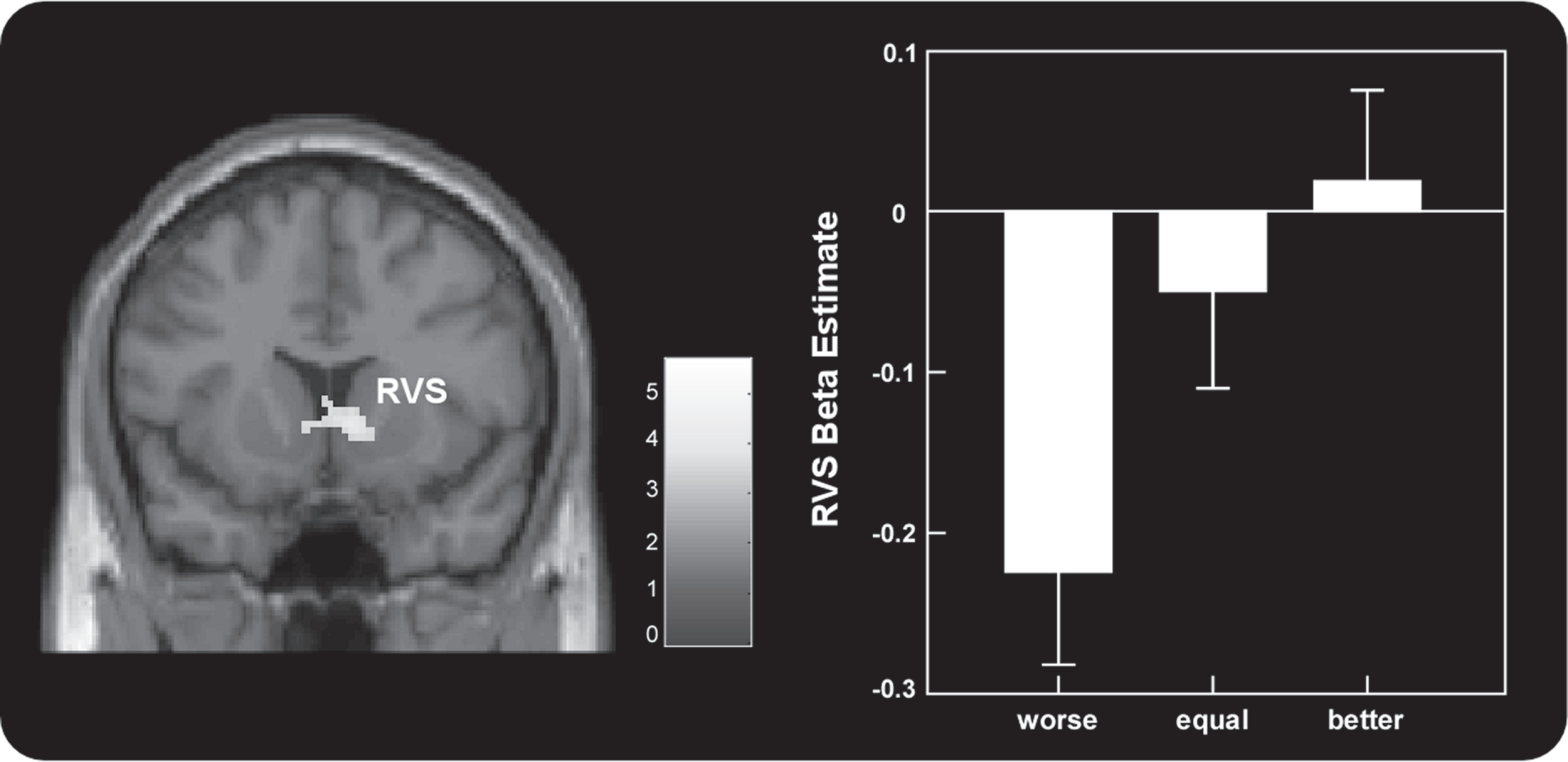
Parametric analyses revealed that the right VS (MNI 8 18 2) showed increased activation as results of social comparison improved. No region showed significant activation as results of social comparison declined.

The right VS (MNI 8 18 2) showed increased deactivation as the results of social comparison deteriorated. Specially, the worse condition revealed strong deactivation in the right VS (Figure 4, Table 2). No regions showed increased activation when results of social comparison got worse.

**Table 2:** The effect of social comparison in Gain trials.

		Peak Activation				
	Region	X	Y	Z	*t* Value	Voxels
***Increased deactivation as results of social comparison deteriorated***					
R	Ventral striatum (VS)	8	18	2	5.68	283
***Increased deactivation as results of social comparison improved***					
	no regions					

Note. Coordinates (mm) are in MNI space. L = left hemisphere; R = right hemisphere. All the clusters survived FWE correction (*P* < 0.05) for multiple comparisons at the cluster level with a voxel-level threshold corresponding to *P* < 0.0001 uncorrected.

**Fig. 5:**
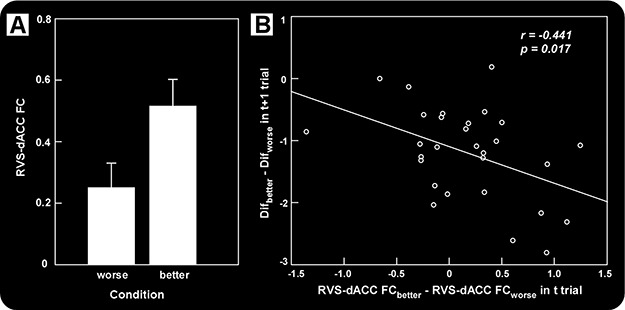
PPI analyses revealed that the right VS (MNI 8 18 2) showed significantly higher functional connectivity with the dACC (MNI −6 24 30) in the better condition compared to the worse condition (A). Moreover, the change of functional connectivity between the VS and the dACC across worse *vs* better condition was negatively correlated with the difference of behavioral changes (Dif) between the two conditions (B).

To investigate how functional connectivity across brain regions during regret processing varied along different levels of social comparison, whole-brain PPI analyses were performed to examine how functional connectivity between the right VS (MNI 8 18 2), identified in the above analysis, and other brain regions varied with social comparison. Results revealed that the right VS showed significantly higher functional connectivity with the dACC (MNI −6 24 30), pregenual anterior cingulate cortex (pgACC; MNI 2 48 8) and the thalamus (−18 −24 14) in the better condition as compared to the worse condition (Figure 5A, Table 3). No other significant effects were found. Interestingly, the change in functional connectivity between the VS and the dACC across worse *vs* better conditions was negatively correlated with the difference in behavioral changes (Dif) between the two conditions (*r* = −0.441, *P* = 0.017) (Figure 5B). More specifically, if the functional connectivity between the VS and the dACC of one participant was more sensitive to social comparison, the subsequent risk-taking behavior of him or her was more affected by social comparison.

**Table 3:** Brain regions showed stronger functional connectivity with right VS in better condition relative to worse condition

		Peak Activation				
	Region	X	Y	Z	*F value*	Voxels
L	Cerebellum	−22	−70	−18	9.49	14 188
R	Precentral	38	4	52	10.08	2890
L	Thalamus	−18	−24	14	8.12	2085
L	VS	−14	16	10	7.26	
R	PgACC	2	48	8	6.60	892
R	DACC	8	36	24	6.36	
R	Inferior temporal gyrus	56	−30	−14	7.66	341
R	Middle frontal gyrus	30	56	32	6.96	107

Coordinates (mm) are in MNI space. L = left hemisphere; R = right hemisphere. All the clusters survived FWE correction (*P* < 0.05) for multiple comparisons at the cluster level with a voxel-level threshold corresponding to *P* < 0.0001 uncorrected.

#### The effect of obtained outcome in Gain trials

**Fig. 6:**
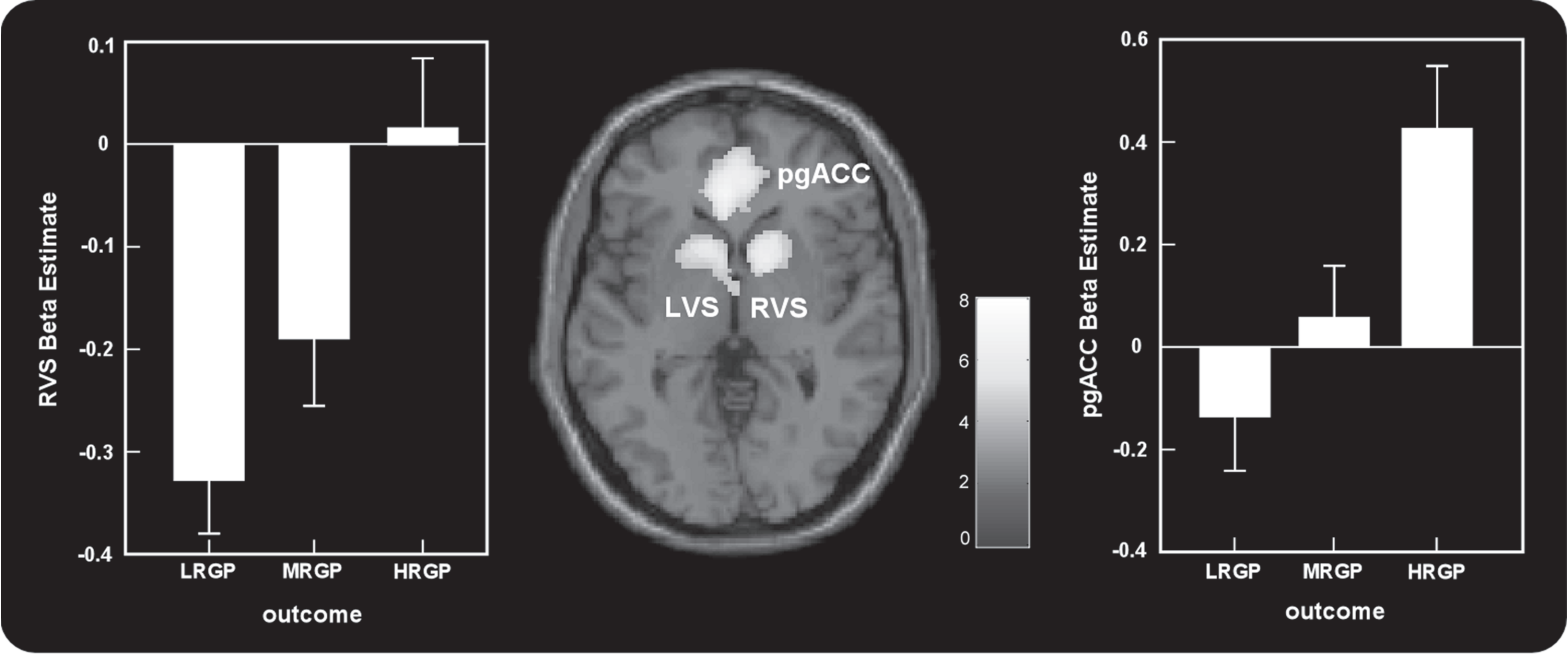
Parametric analyses revealed bilateral VS (MNI 14 8 −4 and −10 10 −2) and the pgACC (MNI 2 46 4) showed increased activation as objective outcomes improved (from LRGP to HRGP). In addition, no regions showed significant activation as objective outcomes declined.

Bilateral VS (MNI 14 8 −4 and −10 10 −2) and the pgACC (MNI 2 46 4) showed increased activation as RGP levels increased (Figure 6, Table 4). Moreover, the LRGP outcome showed strong deactivation in the VS. In addition, no regions showed increased activation as the level of RGP decreased.

**Table 4:** The effect of outcomes in Gain trials

		Peak Activation				
	Region	X	Y	Z	*t value*	Voxels
***Increased with increasing RGP level***					
R	PgACC	2	46	4	7.99	1731
R	MCC	4	−32	44	6.94	1189
L	MCC	−2	−20	40	5.09	
R	VS	14	8	−4	4.01	941
L	VS	−10	10	−2	5.88	580
L	Middle occipital gyrus	−26	−94	4	5.05	434
R	Calcarine gyrus	26	−92	2	5.75	306
L	Supramarginal gyrus	−48	−42	32	4.89	258
R	Supramarginal gyrus	64	−38	36	6.18	161
L	Superior frontal gyrus	−14	36	36	6.35	145
***Increased with decreasing RGP level***					
No regions					

Coordinates (mm) are in MNI space. L = left hemisphere; MCC = middle cingulate cortex; R = right hemisphere. All the clusters survived FWE correction (*P* < 0.05) for multiple comparisons at the cluster level with a voxel-level threshold corresponding to *P* < 0.0001 uncorrected.

**Fig. 7:**
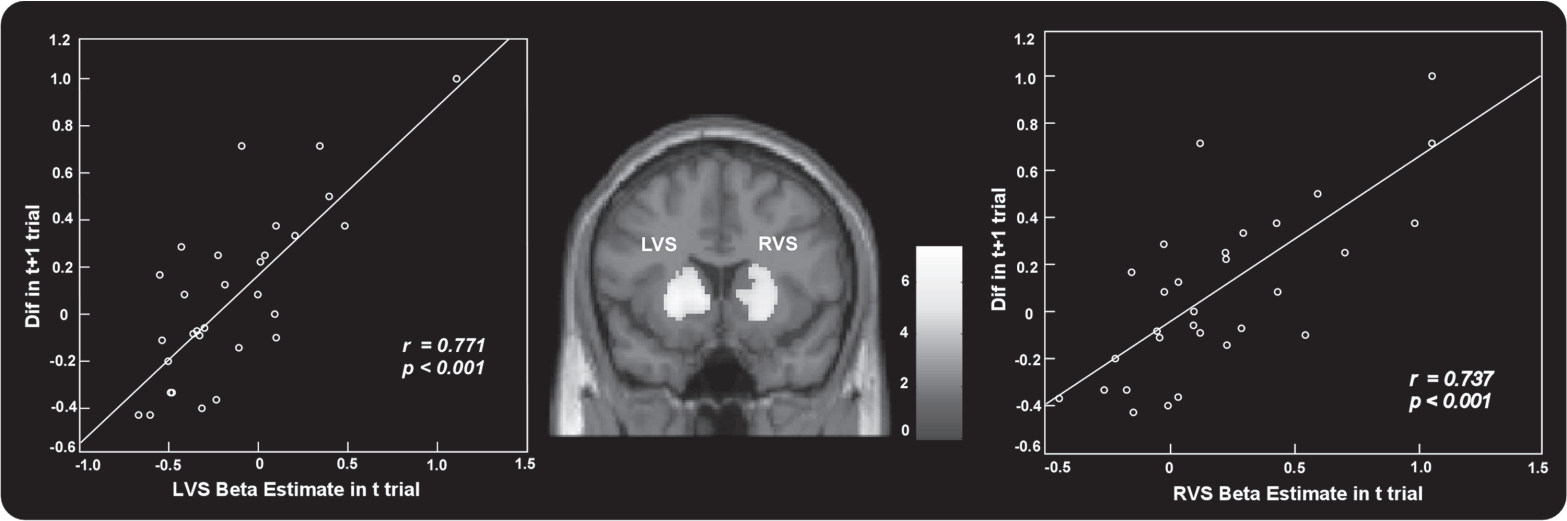
In MRGP outcome, the results revealed that activations of the left VS (MNI −10 16 0) and the right VS (MNI 24 12 −4) in the current trial were positively correlated with corresponding behavioral changes in the next trial across participants.

To investigate the relationship between neural response to regret and the succeeding behavioral changes (Dif), the brain–behavior correlation analyses across participants were conducted for the HRGP outcome, the MRGP outcome and the LRGP outcome. Only in the MRGP outcome did the results reveal that activation of the bilateral VS (MNI −10 16 0 and 24 12 −4) was positively correlated with corresponding behavioral changes across participants (Figure 7).

#### The effect of emotional rating in Gain trials

The left dorsal striatum (MNI −18 8 24), pgACC (MNI −6 44 8), medial prefrontal cortex (mPFC) (MNI −12 50 6) and the right VS (MNI 12 22 2, albeit at a more lenient threshold) showed increased activation as emotional ratings increased (Table 5). No regions showed significant activation as emotional ratings decreased. In addition, in Loss trials, no regions showed significant activation with increasing or decreasing emotional ratings.

**Table 5:** The effect of emotional rating in Gain trials

		Peak Activation				
	Region	X	Y	Z	*t value*	Voxels
***Increased with increasing emotional rating***					
L	dorsal striatum	−18	8	24	4.62	290
L	pgACC	−6	44	8	5.05	263
L	*mPFC*	−12	50	6	4.88	
***Increased with decreasing emotional rating***					
No regions					

Coordinates (mm) are in MNI space. L = left hemisphere; R = right hemisphere. All the clusters survived FWE correction (*P* < 0.05) for multiple comparisons at the cluster level with a voxel-level threshold corresponding to *P* < 0.0001 uncorrected.

#### The effect of social comparison in Loss trials

Loss trials had two conditions: worse and equal. The equal–worse contrast activated bilateral VS (MNI 12 20 −2 and −14 20 2). The reverse contrast did not show suprathreshold activation (Table 6).

#### The effect of lost coins in Loss trials

In Loss trials, the value of RGP was zero so the number of lost coins was used as the parametric regressor. Bilateral VS (MNI 8 10 −4 and −10 20 −6) showed increased activation as the number of lost coins decreased. No regions showed significant activation as the number of lost coins increased. (Table 7).

## Discussion

In the present study, we employed a modified sequential risk-taking task to investigate the modulation of social comparison on the neural responses to regret and subsequent risk-taking behavior. Behaviorally, the results showed that emotional ratings and risk-taking behavior were affected by both social comparison and obtained outcomes. Specifically, participants felt less regret and took less risk when results of social comparison were positive (from worse to better condition) or when objective outcomes improved (from LRGP to HRGP outcome). Moreover, the results showed that the more regret participants experienced in the current trial, the more risks they would take in the next trial, which is consistent with our previous work (Liu *et al.*, 2016). At the neural level, as levels of RGP increased, increased activation of the VS and the pgACC was found. Specifically, in the MRGP outcome, the results revealed that activation of the VS was positively correlated with corresponding behavioral changes across participants. Moreover, increased activation of the VS accompanied the improvement of social comparison. Interestingly, the right VS showed significantly higher functional connectivity with the dACC and the pgACC in the better condition, compared to the worse condition. In addition, the change of functional connectivity between the VS and the dACC in the worse condition and better condition was negatively correlated with the difference of Dif (difference in behavioral changes across successive trials) in both conditions.

**Table 6:** The effect of social comparison in Loss trials

		Peak Activation				
	Region	X	Y	Z	*t value*	Voxels
***Equal–worse***					
L	Precentral	−28	−24	58	7.02	4496
L	Calcarine gyrus	−10	−88	12	7.20	3578
R	VS	12	20	−2	8.69	3470
L	VS	−14	20	2	7.70	
R	Middle temporal	44	−66	6	6.25	868
L	Superior frontal gyrus	−26	30	34	6.18	596
R	Temporal pole	58	12	−12	4.83	420
R	Hippocampus	36	−26	−12	5.76	267
L	Superior frontal gyrus	−22	10	62	5.13	161
***Worse–equal***					
No regions					

Coordinates (mm) are in MNI space. L = left hemisphere; R = right hemisphere. All the clusters survived FWE correction (*P* < 0.05) for multiple comparisons at the cluster level with a voxel-level threshold corresponding to *P* < 0.0001 uncorrected.

**Table 7:** The effect of lost coins in Loss trials

		Peak Activation				
	Region	X	Y	Z	*t value*	Voxels
***Increased with decreasing number of lost coins***					
L	Precentral	−22	−18	58	6.31	697
R	VS	8	10	−4	5.53	543
L	VS	−10	20	−6	5.21	
L	MCC	−10	−28	46	5.05	149
***Increased with increasing number of lost coins***					
No regions					

Coordinates (mm) are in MNI space. L = left hemisphere; MCC = middle cingulate cortex; R = right hemisphere. All the clusters survived FWE correction (*P* < 0.05) for multiple comparisons at the cluster level with a voxel-level threshold corresponding to *P* < 0.0001 uncorrected.

In line with our hypothesis, the experience of regret and regret-based learning were modulated by not only alternative outcomes that might be achieved by one’s self but also by outcomes achieved by others. The current results replicated our previous findings that participants felt less regret with increasing RGP levels and participants took more risk after stronger feelings of regret (Liu *et al.*, 2016). The results also supported previous accounts where participants learned from past experience of regret to direct future behavior (Coricelli *et al.*, 2005; Lohrenz *et al.*, 2007; Chiu *et al.*, 2008; Marchiori and Warglien, 2008). Previous studies have investigated the effects of social comparison on decision-making and emotions (Crosby, 1976; Wheeler and Miyake, 1992; Collins, 1996; He, 1997; Kumar, 2004; Hoelzl and Loewenstein, 2005; Linde and Sonnemans, 2012). For example, Collins (1996) proposed that comparisons with others who are better off than oneself can sometimes produce negative emotions, such as resentment and depression. Moreover, previous studies found that individuals took advantage of comparisons to obtain knowledge that may be more effectively deployed in similar future situations (Buunk *et al.*, 1990; Testa and Major, 1990). Specifically, Bault *et al.* (2008) proposed an interdependent utilities model, which indicated that social comparison could affect emotions and future behavior. They found that individuals learned from social comparison and evaluated past outcomes to adjust choices in the future. In agreement with previous researchers and the interdependent utilities model, our results showed that participants felt more regret and took more risk in the better condition compared to the worse condition.

In the current study, the VS showed increased activation as RGP levels increased. Specifically, previous studies that employed similar sequential risk-taking tasks have observed stronger activation of the VS in the optimal outcome. The results suggested that increased activation in the VS with increasing RGP levels reflected their roles in the ‘reward system’, which has been repeatedly identified during decisions involving rewards (Rogers *et al.*, 2004; Marsh *et al.*, 2007; Izuma *et al.*, 2008; Haber and Knutson, 2010). Moreover, far more explicit models have argued that the VS is involved in reward-related prediction error (the difference between expected and obtained outcomes) (Schultz, 2016). Our previous work (Liu *et al.*, 2016) revealed that the tipping point between reporting regret *vs* relief was approximately an RGP of three-fifth. In other words, participants felt no regret or relief when they encounter an RGP of 0.6. We considered that there might be a negative prediction error if 0 < RGP < 0.6, i.e. LRGP is a form of a strong negative surprise signal. Concurrently, LRGP outcome showed strong deactivation in the VS that might be reflective of some kind of negative prediction error (i.e. ‘I did worse than I could have’). Studies of prediction error for rewards have shown that outcome omission (i.e. negative prediction error) results in deactivation of the VS. On the other hand, there might be a positive prediction error if 0.6 < RGP < = 1. In optimal outcomes (RGP = 1, unexpected stimulus), the positive prediction error might reach its extreme point, which could also be considered a form of a strong positive surprise signal. Consequently, our results suggest that activity in the VS is a reward signal but may also contain a prediction error.

Moreover, in the MRGP outcome, the results reveal that activation of bilateral VS in the current trial was positively correlated with corresponding behavioral changes in the next trial across participants. This result is one of the novel findings within the current study and extends previous data showing that activation patterns in the brain can predict reversals (Hampton and O'Doherty, 2007; Boorman *et al.*, 2009) and choices in economic gambles (Kuhnen and Knutson, 2005; Venkatraman *et al.*, 2009). Extensive converging evidence indicates a role of the VS in the learning of stimulus–response associations (Knowlton *et al.*, 1996; Jog *et al.*, 1999). These findings emphasise a crucial role for the VS in learning that is based on trial-by-trial feedback to update responses (Delgado *et al.*, 2005; Daw *et al.*, 2006). Furthermore, research has suggested a key role for the VS in learning to modify actions based on predicted outcomes and provided an obvious link between the VS and motivated behavior. Collectively, the VS guides decision-making by integrating value to drive motivated behavior. Intriguingly, the current study found a direct relationship between VS and motivated behavior. In accordance with previous findings, we suggest that the VS, as a key neural structure involved in reward-related processing, plays a crucial role in recognising and evaluating rewards, learning from rewards and predicting the best potential reward in the future (Cools *et al.*, 2002). We considered the reason why the significant correlation between activations of the VS in the current trial and behavioral changes in the next trial were found only in MRGP. An outcome with MRGP was neither excellent (i.e. HRGP outcome) nor extremely poor (i.e. LRGP outcome). It might be reasonable that individual differences between participants would be more effective in changing future behaviors, when the current behavioral status was mild instead of extreme.

The dorsal striatum showed increased activation as emotional ratings increased within each participant. A similar signal was observed in the VS; however, this activation was not significant when correcting for multiple comparisons, and we therefore refrain from interpreting this finding. The behavioral results revealed that participants took more risk after experiencing a high level of regret. These results might enlighten us about the relation between behavioral results, affective ratings and neural processes. However, we did not find a relationship between activation of the dorsal striatum in the *t* trial and behavioral change in the *t* + 1 trial.

Notably, the results showed that activation of the VS was modulated not only by obtained outcome but also by social comparison. Specifically, the VS showed increased activation as a result of social comparison improved. These findings support results from previous studies reporting that the VS encodes social rewards (Izuma *et al.*, 2008, 2010) and positive social comparison (Fliessbach *et al.*, 2007). Interestingly, the VS showed strong deactivation when participants performed worse than others. Previous evidence has demonstrated a pattern of VS activation like this in social competition when participants lose an auction to another person (Delgado *et al.*, 2008). The finding of deactivation in VS might reflect the negative prediction error in a social context (i.e. ‘I did worse than others’). Moreover, the VS showed significantly higher functional connectivity with the dACC and the pgACC in the better condition as compared to the worse condition. The results suggest that positive social comparison enhances both activation of the VS and functional connectivity within the ‘reward system’. In addition, the results also showed the change of striatum–dACC functional connectivity between the worse condition and better condition was negatively correlated with the difference of Dif in both conditions, across participants. In other words, when participants’ functional connectivity between the VS and the dACC was more sensitive to the results of social comparisons, greater difference in the tendency to take risks was observed. Previous neuroimaging studies have considered the functional connectivity between the VS and the dACC as vital to risk-taking behaviors (Haber and Knutson, 2010; Porter *et al.*, 2015). Porter *et al.* (2015) proposed that motivation, carried by the VS, tightly influences dACC activity, which may be implicated in the propensity for adolescents to engage in risk-taking behavior. The current results might be helpful in understanding the specific role of the dACC in learning value and the interacting relationship with the VS to guide future actions.

Previous work using a similar risk-taking task has found that activations of the pgACC and the mPFC were sensitive to obtain outcome and emotional rating in non-social conditions (i.e. when the other person’s outcome is not revealed). Consistent with previous findings, the current study demonstrated that the pgACC and the mPFC showed increased activation with both increasing RGP level and increasing emotional rating and extended this work to show that activations of the pgACC and the mPFC were not modulated by social comparison. Previous studies revealed that self-reflection and person perception were associated with activity extending from the anterior cingulate cortex to the mPFC (Amodio and Frith, 2006). For example, Kelley *et al.* (2002) observed more activity in the mPFC of participants when they were thinking about attributes of the self *vs* other people. Moreover, Steele and Lawrie (2004) have suggested that this region is concerned with self-reported emotion. The current study therefore suggested that activations of the pgACC and the mPFC were associated with absolute rewards directly achieved by one’s self, not by rewards in comparison to others.

Bault *et al.* (2011) investigated the neural underpinnings of the effect of social comparison on risky choices by using a modified version of the ‘wheels of fortune’ task (Camille *et al.*, 2004; Coricelli *et al.*, 2005; Habib *et al.*, 2015). In this task, the actual outcome of participants could be divided into a gain or loss outcome. They found that social comparison modulated activation of the VS and future behavior. Specifically, experiencing social gains induced more risky and competitive behavior in later trials and the VS showed higher activity for social gain relative to social loss. Additionally, the activity of the striatum during the outcome evaluation of the current trial was correlated with the mPFC activity in the choice period of the next trial. In the current study, we investigated the effect of social comparison during the sequential risk-taking task. In line with Bault *et al.*’s findings, the present results revealed that social comparison indeed could modulate activation of the VS and future behavior. As well as these consistent results, the current study also found some unique results. Firstly, the functional connectivity between VS and prefrontal regions during the evaluation of outcome was affected by social comparison. Specifically, the VS showed significantly higher functional connectivity with the dACC in the better condition as compared to the worse condition. In addition, the VS–dACC functional connectivity was related to future risk-taking behavior. These results extend previous findings and indicate that not only activation of the VS but also its functional connectivity is affected by the state of social comparison. Secondly, only in MRGP (i.e. a moderate outcome status instead of an extremely good or bad one) were activations of bilateral striatum in the current trial positively correlated with corresponding behavioral changes in the next trial. The results might have indicated that the prediction of the VS on future behavior was modulated by outcome status.

In summary, by using the modified sequential risk-taking task, the present fMRI study showed that participants felt less regret and took less risk either when objective outcomes improved or when result of social comparison was more positive. Moreover, in line with our previous work, the current results showed participants tended to take more risk after experiencing regret, which could be termed as regret-based learning. At the neural level, increased activations of the VS and the pgACC were found to correspond with improved objective outcomes. Specifically, activations of the VS were positively correlated with corresponding behavioral changes across participants. As results of social comparison improved, increased activation of the VS was found. With regards to functional connectivity being modulated by social comparison, the VS showed significantly higher functional connectivity with the dACC and the pgACC in the better condition as compared to the worse condition. In addition, the change in functional connectivity between the VS and the dACC in the worse condition and better condition was negatively correlated with the difference of Dif in both conditions.

The main limitation of the current study was that the current research design did not allow the investigation of possible interactions between outcome and social comparison. This was due to the restricted number of experimental trials in each condition. For instance, when participants had an optimal outcome in a trial (RGP = 1), results of social comparison could only be equal to or better than the other. Because of those optimal trials for the HRGP outcome condition, few trials could be identified with a social comparison result worse than the other. Subsequently, we were unfortunately unable to conduct the 3 (outcome: LRGP, MRGP *vs* HRGP) × 3 (social comparison: worse, equal *vs* better) repeated measures analysis of variance with the current task. The present study only investigated the results of social comparison (worse, equal and better) and its modulating effect on regret and subsequent risk-taking behavior. Another limitation of the current research was that the experimental design did not set non-social conditions (i.e. in which the other person’s outcome is not revealed) so we could not compare social conditions and non-social conditions directly. We will investigate this issue in our future work.
